# Copeptin as a New Blood-Based Marker for Differential Diagnosis in Stroke Patients

**DOI:** 10.3390/ijms26115328

**Published:** 2025-06-01

**Authors:** Antonia Ioana Vasile, Sorin Tuță, Cristina Tiu, Corin Badiu

**Affiliations:** 1 Doctoral School, Carol Davila University of Medicine and Pharmacy, 030167 Bucharest, Romania; antoniaioana97.vasile@gmail.com; 2 Neurology Department, University Emergency Hospital of Bucharest, 050098 Bucharest, Romania; cristina.tiu@umfcd.ro; 3 Clinical Neurosciences Department, Carol Davila University of Medicine and Pharmacy, 030167 Bucharest, Romania; 4 National Institute of Neurology and Neurovascular Diseases, 077160 Bucharest, Romania; 5 Endocrinology IV Department, Parhon National Institute of Endocrinology, 011863 Bucharest, Romania; corin.badiu@umfcd.ro

**Keywords:** copeptin, stroke, ischemic stroke, hemorrhagic stroke, blood-based marker, differential, stroke prognosis

## Abstract

Diagnosis in stroke patients is based mainly on clinical and radiological findings; therefore, there is a need for serological markers that can orient the clinician. Copeptin is a new blood marker for diagnosis and prognosis in several neurological conditions, such as ischemic stroke, hemorrhagic stroke, aneurysmal subarachnoid hemorrhage, and multiple sclerosis. The aim of our study was to highlight the diagnostic value of copeptin in differentiating between stroke subtypes and stroke mimics. We performed a literature review by searching the PubMed and Scopus databases for papers with the following keywords: “stroke AND copeptin AND differential”. The PRISMA criteria were used. We identified 29 papers that met the criteria. We analyzed only original research articles, excluding reviews and only including those in English. Some studies did not find any significant differences between cerebral infarction, intracerebral hemorrhage, and subarachnoid hemorrhage, but one study demonstrated significant correlations. All studies agreed that copeptin levels can help in differentiating stroke patients from stroke-free patients. Copeptin levels were correlated with prognostic scales For stroke mimics, copeptin levels were extremely broad and for vestibular disorders; it was shown that a normal level of copeptin excludes stroke. Copeptin is a new blood marker that can help clinicians in the acute neurological field. It may help in diagnosing stroke, in differentiating between stroke subtypes and stroke mimics, and in evaluating the prognosis of these patients, but further studies are needed.

## 1. Introduction

Despite significant advances in the management of stroke patients, there is still a lack of serological markers that can direct the diagnosis of these patients.

The clinical diagnosis of stroke in the emergency room can be difficult and often necessitates brain imaging. CT and MR imaging are the only paraclinical evaluations capable of excluding hemorrhagic stroke or other conditions that resemble a stroke. However, there are certain situations, like the unavailability of the radiology department, where time-sensitive decisions to initiate revascularization treatment (thrombolysis and mechanical thrombectomy) rely solely on the clinical presentation [1]. In addition, there are multiple centers in rural areas in developing countries where radiology departments do not perform routine CT examinations or have no CT machine at all. Moreover, there are situations in emergency hospitals where there is a need to diagnose a stroke versus a non-neurological disease for triage situations, admission reasons, or therapeutic management (for example, a comatose patient where the cause of the coma may be a metabolic disorder or a vertebro-basillary stroke). In these situations, blood-based markers that may orient the diagnosis and differentiate stroke could be helpful.

Research indicates that higher copeptin levels are associated with prognosis and can help in the differential diagnosis before imaging in several cardiovascular conditions, including acute myocardial infarction, congestive heart failure, ischemic stroke, aneurysmal subarachnoidal hemorrhage, and head trauma [2,3,4,5,6]. Moreover, copeptin has recently gained significant attention in the neurological field [1,7,8,9,10,11]. It was previously shown that copeptin is a good diagnostic marker for several neurological conditions, such as ischemic stroke, nontraumatic intracerebral hemorrhage, aneurysmal subarachnoid hemorrhage, and multiple sclerosis [12].

**Figure 1:**
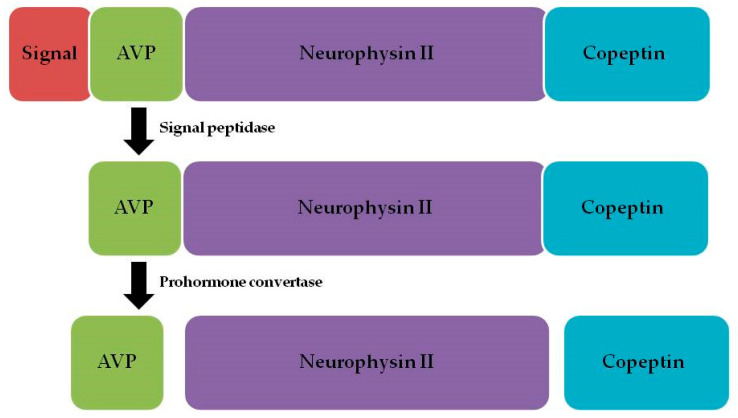
Pro-arginine vasopressin and its components.

Copeptin is one of the three neuropeptides that form pro-arginine vasopressin (pro-AVP), alongside argininevasopressin and neurophysin II [13] (Figure 1). 

Arginine vasopressin (AVP), or antidiuretic hormone (ADH), is released by the neurohypophysis in response to stress, elevated plasma osmolality, or a reduced blood volume [14]. However, the physiological role of AVP is to maintain fluid balance, regulate vascular tone, and modulate the endocrine response to stress, while the specific physiological function of copeptin is yet to be determined [10]. Previous studies have suggested that AVP plays an important role in regulating the brain volume, electrolyte homeostasis, and microvascular resistance in ischemic stroke [15]. This finding suggests that dysfunction in AVP secretion may explain the severity and prognosis of ischemic stroke patients.

Copeptin can be identified in circulation in equimolar amounts with AVP [16,17,18]. Copeptin is a new biomarker formed by a 39-amino-acid-long glucosylated peptide with a leucine-rich core region [10]. Because both copeptin and AVP are processed from the same precursor peptide, the release of both is regulated by the same stimuli: an increase in systemic osmolality and a decrease in the blood volume and pressure [19].

Copeptin measurement is easier compared to measuring AVP because copeptin has the advantage of being a more stable molecule and is bound to platelets [10,20,21,22]. The half-life of AVP is reported to be between 10 and 44 min, while the half-life of copeptin is two times longer; the difference is based on their distinct metabolic clearance rates [10]. Copeptin measurement requires only a small sample blood volume and no extraction step, and the sample is more stable even up to 7 days after sampling if stored at room temperature and up to 14 days if stored at 4 degrees [10,23].

Copeptin can be measured with two commercially available assays that are widely available and validated: a manual sandwich immunoluminometric assay and an automated immunofluorescent assay [10]. In addition, a serum copeptin assay can yield results within less than 2 h, which makes it a useful tool for emergency room dosing [10,24]

The normal range of copeptin has been assessed in several studies: the first study included more than 300 subjects with levels that varied between 1.0 and 13.8 pmol/L (with a mean of 4.2 pmol/L), while the second study included more than 700 subjects with levels that varied similarly between 1.0 and 13.0 pmol/L [10,17,19].

When evaluating ischemic stroke patients, increased copeptin levels are correlated with the functional outcome and all-cause mortality within 3 months, 6 months, and 1 year after acute ischemic stroke [25]. Higher copeptin levels are significantly elevated in stroke patients compared to healthy controls. Moreover, higher levels of copeptin are associated with increased stroke severity, as calculated by NIHSS scores, and are correlated with higher mortality rates, higher disability rates, and poorer clinical outcomes, as calculated by the mRS score and Barthel index in previous studies [10,26]. Moreover, the prognostic accuracy of copeptin in stroke patients was found to be greater than that of other frequently assessed laboratory parameters, including blood glucose, C-reactive protein, and white blood cell counts, in addition to clinical indicators such as blood pressure and temperature [27].

However, most of the studies that have assessed copeptin in stroke patients have focused on ischemic stroke and the prognostic value of this marker for stroke severity, treatment efficacy, stroke complications, functionality, mortality, and recurrence [25].

Only a few studies have focused on how copeptin levels can guide the differential diagnosis of stroke. To this aim, the present study presents a review of recent studies that have assessed the diagnostic value of the plasma copeptin concentration in differentiating between subtypes of stroke and stroke mimics.

## 2. Materials and Methods

We performed a review of the literature, analyzing the PubMed and Scopus databases. The search was limited to articles in the English language. We applied a search filter to obtain papers published between the years 2010 and 2024. The PRISMA criteria were used [28].

The first search used the keywords ”stroke”, ”copeptin”, and ”differential”. The PubMed database yielded 16 results. The Scopus database yielded 13 results.

**Figure 2:**
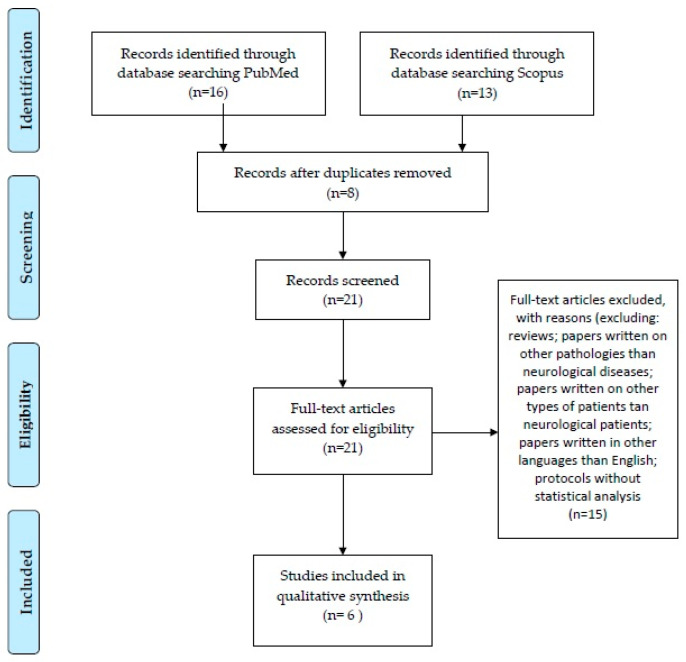
PRISMA diagram.

After excluding duplicate articles between the two databases (n = 8), 21 articles were obtained for analysis. We focused on original research papers, excluding reviews (n = 4). We also focused on neurological diseases, excluding papers written on other pathologies (hyponatremia, heart failure, type 2 myocardial infarction, acute diseases, hyponatremia; n = 6) or other types of patients (pregnant patients, recreational marathon runners; n = 2). We also analyzed only articles written in English (excluding one paper in German). We also excluded articles that presented only protocols, without a statistical analysis (n = 2). In the end, the two databases resulted in 6 articles that answered our research question. The PRISMA diagram can be consulted in Figure 2.

## 3. Results

### 3.1. Copeptin May Differentiate Between Stroke Subtypes

Ischemic stroke, intracerebral hemorrhage, and subarachnoid hemorrhage are cerebrovascular conditions that are observed in the emergency department with varied clinical presentations [7]. Radiological imaging (CT or MRI) is used for definitive diagnosis. In this situation, finding a biochemical parameter that differentiates between severe cerebrovascular pathologies may help with treatment prioritization, intensive care unit admission requirements, and survival rate estimates, especially in medical units that have limited access to radiology departments.

Aksu et al. (2016) [7] assessed whether copeptin could help in the differential diagnosis of cerebral infarction, intracerebral hemorrhage, and subarachnoid hemorrhage in the emergency room. They obtained blood samples to determine the copeptin level before imaging for 176 patients. However, in their study, the serum level of copeptin was calculated retrospectively: five-millimeter blood samples were obtained from the patients and centrifugated, but the plasma was stored at −70 degrees and the lab result was obtained later during hospitalization. Thus, they identified a copeptin level of 5.49 ng/dL for cerebral infarction; 4.50 ng/dL for intracerebral hemorrhage; 5.90 ng/dL for subarachnoid hemorrhage; and 2.0 ng/dL for healthy volunteers. There was no significant correlation between the copeptin level and the intracerebral hemorrhage score (ICH). However, significant correlations were identified with the NIHSS score and Hunt and Hess score. In addition, the copeptin levels in patients who died were higher compared to those who were discharged. A negative correlation between the copeptin level and Glasgow Coma Scale (GCS) was also identified. Although healthy volunteers were statistically found to have lower levels of copeptin compared to those with neurological pathologies, there appeared to be no significant difference in the copeptin levels between cerebral infarction, intraparenchymal hemorrhage, and subarachnoid hemorrhage. In conclusion, the researchers could not statistically highlight that copeptin levels help to differentiate between these three neurological pathologies.

CT and MRI are the most widely used to confirm the diagnosis of stroke and to differentiate between ischemic and hemorrhagic stroke, but imaging is not available easily across all hospitals, especially in rural areas. Han et al. (2023) [29] analyzed several markers to help differentiate between ischemic stroke and hemorrhagic stroke. They evaluated several markers in 73 patients with acute stroke-like symptoms, of which 51 had ischemic stroke and 22 had hemorrhagic stroke. However, in their study, the serum level of copeptin was calculated retrospectively: blood samples were obtained and centrifuged, but the plasma samples were aliquoted into cryotubes and stored at -80 degrees, and the lab result was obtained later. They demonstrated no significant intergroup differences in the copeptin concentration (median of 41.1 pmol/L for ischemic stroke and 41.9 pmol/L for hemorrhagic stroke). Their conclusion was that plasma glial fibrillary acidic protein (GFAP) and NT-proBNP can help in the differential diagnosis between ischemic and hemorrhagic stroke. Moreover, they explained that using a combination of GFAP and NT-proBNP is a feasible strategy for differentiation between subtypes in the hyperacute phase. GFAP is an intermediate filament found only in astrocytes [30]. The differential levels of GFAP between ischemic and hemorrhagic stroke can be attributed to the different kinetics of astrocytic cell death. Necrosis appears in the first few hours of symptom onset for hemorrhagic stroke, while astrocytic cell death peaks at 48–98 h after symptom onset for ischemic stroke [31].

Sun et al. (2018) [32] evaluated 119 healthy versus 119 stroke patients (87 with ischemic stroke and 32 with hemorrhagic stroke) from rural China. This was a 1:1 case–control study that investigated whether copeptin could indicate the etiological factor of the stroke. However, in their study, the serum level of copeptin was calculated retrospectively: venous blood was obtained from the patients and centrifuged immediately, but the plasma was stored at -80 degrees and the lab result was obtained later. The mean level of copeptin in the group of patients with ischemic stroke was 20.90 pmol/L; that in the group of patients with hemorrhagic stroke was 6.53 pmol/L; and that in the group of healthy subjects was 8.42 pmol/L. They demonstrated that the copeptin levels were higher in stroke patients compared to healthy controls (20.9 pmol/L versus 8.42 pmol/L, with a statistically significant difference). The conclusion of this study was that the copeptin level was positively associated with ischemic stroke and negatively associated with hemorrhagic stroke [32]. The authors suggested that copeptin might be a meaningful marker for differentiation between ischemic and hemorrhagic stroke and plays a potential role in the arginine vasopressin system in the pathophysiology of both types of stroke [32]. Moreover, copeptin could be used as one of the combined markers to improve the accuracy of the early identification of stroke subtypes [32].

Sarfo et al. (2018) [33] evaluated three markers (GFAP, copeptin, and matrix metalloproteinase-9 (MMP-9)) in 156 stroke patients and 74 stroke-free patients. Here, 99 of the patients had ischemic strokes and 57 had hemorrhagic strokes. However, in their study, the serum level of copeptin was calculated retrospectively: five-millimeter blood samples were obtained from the patients and centrifuged, but the plasma was stored at −80 degrees until analysis. The copeptin levels were higher among stroke patients compared to healthy controls (mean of 21.2 pmol/L compared to 10.7 pmol/L). The authors did not find any differences between ischemic stroke patients and hemorrhagic stroke patients in the concentrations of any of the three markers. They suggested that this result was due to the late presentation of patients to the hospital (a mean of 6 days for hemorrhagic stroke and 7 days for ischemic stroke). However, when they restricted the analysis only to patients who presented within 3 days of symptom onset, copeptin was marginally higher among ischemic stroke subjects compared to hemorrhagic stroke patients (26.3 pmol/L compared to 20.7 pmol/L). They also observed that the GFAP levels declined among the three etiologies of strokes, being the highest for large-vessel atherothrombotic stroke, followed by cardioembolic strokes and then lacunar strokes. This difference was not observed for copeptin or MMP-9. The authors did not find any significant correlations between the lesion volume and the three evaluated markers. However, copeptin was negatively correlated with stroke severity. The three markers did not differ between patients who died and patients who survived. None of the markers were associated with stroke severity or mortality. None of the markers distinguished between ischemic and hemorrhagic stroke within 3 days of stroke onset. However, GFAP and copeptin were excellent markers in differentiating between stroke patients and stroke-free patients [33].

On the other hand, an increased plasma copeptin level was demonstrated as an independent prognostic marker of 1-year mortality and 1-year unfavorable outcomes for intracerebral hemorrhage [34]. The prognostic value of copeptin to predict 30-day mortality and functional outcomes after 90 days for intracerebral hemorrhage was also confirmed [35].

Aneurysmal subarachnoid hemorrhage (aSAH) is a cerebrovascular event with long-term morbidity and mortality [36]. Delayed cerebral ischemia, which can happen between 4 and 14 days after the onset of aSAH, is the most important determinant of a poor outcome [37]. Zuo and Ji (2019) [38] analyzed 243 patients with aSAH and evaluated mortality and poor functional outcomes (measured on the Glasgow Outcome Scale) after 3 months. The median copeptin level was 21.0 pmol/L. Copeptin levels were higher when the severity of aSAH was higher (defined by the WFNS score). The mean copeptin level was 17.2 pmol/L for patients with a WFNS score below 3, whereas it was 29.6 pmol/L for patients with a WFNS score above 3. Patients who experienced a poor outcome at the 3-month follow-up had a level of copeptin that was nearly two times higher than in patients who experienced a good outcome (mean of 30.7 pmol/L compared to 19.0 pmol/L). Patients with poor outcomes and patients who died had significantly higher levels of copeptin at admission. The researchers demonstrated that the factors that predicted the 3-month outcome were age, WFNF at admission, the modified Fischer score at admission, intraventricular hemorrhage, delayed cerebral ischemia, the level of IL-6, and the level of copeptin. Based on the ROC curve, they demonstrated that a cutoff of 24.0 pmol/L for copeptin serves an indicator for the prediction of poor outcomes, which demonstrated sensitivity of 70.5% and specificity of 69.6%. In addition, when analyzing the discriminatory ability to predict poor outcomes, the model containing copeptin as a factor plus the known risk factors showed a greater discriminatory ability compared to the model without copeptin [38]. Patients who died had a level of copeptin that was more than two times higher than in patients who survived (mean of 47.8 pmol/L compared to 19.9 pmol/L). Based on the ROC curve, copeptin combined with the WFNS score had greater discriminatory accuracy compared to the WFNS score alone [38]. For the patients with good outcomes (who truly returned to normal daily function), the plasma copeptin levels were *lower* compared to patients with poor outcomes (20.5 pmol/L compared to 30.1 pmol/L) [38]. Zuo and Ji (2019) [38] concluded that the copeptin level can predict the short-term prognosis (mortality and functionality) from the moment of onset in aSAH patients. Another important conclusion of their study was that copeptin can improve the prognostic accuracy of the WFNS score. Fung et al. (2013) [39] reported that copeptin can indicate the clinical severity of the initial bleeding in aSAH and, therefore, may guide treatment decisions. They evaluated 18 patients with aSAH and observed correlations between the levels of copeptin and the severity of aSAH as measured by the *WFNS* score after resuscitation, the amount of subarachnoid blood, and the occurrence of intracerebral hemorrhage. In their study, the median copeptin level at admission was 17.0 pmol/L. Dividing them according to the WFNS score, the median copeptin levels were 6.8 pmol/L for WFNS 1, 2.8 pmol/L for WFNS 2, 7.1 pmol/L for WFNS 3, 17.4 pmol/L for WFNS 4, and 79.9 pmol/L for WFNS 5. The authors also found an association between higher levels of copeptin and poor-grade aSAH (based on WFNS 4–5 points), but also the existence of intracerebral hemorrhage. Moreover, the copeptin levels of patients who had a good outcome were lower than in those with poor outcomes, suggesting that copeptin levels may predict outcomes for patients with aSAH. Moreover, patients who died had median copeptin levels that were three times higher than in patients who survived at the 6-month follow-up, explaining that the copeptin levels may predict mortality for patients with aSAH. However, there were no significant correlations among copeptin and the aneurysm location, intraventricular hemorrhage, hydrocephalus, vasospasm, or delayed cerebral ischemia. This result indicates that copeptin levels should not be used as a predictor for vasospasm because clinical grades have higher accuracy [39,40].

### 3.2. Copeptin May Differentiate Between Stroke Mimics

Deboevere et al. (2019) [41] evaluated whether copeptin and PS100 levels can help in the differential diagnosis between stroke and other causes of vertigo in the emergency room. It is always a challenge for the attending physician to exclude the diagnosis of stroke (which would require emergency neurovascular treatment) from other peripheral causes of vertigo (where treatment is usually only symptomatic). Protein S-100b (PS100) is a marker of vascular or traumatic brain injury [42]. It is a protein secreted by central nervous system astrocytes and has negative predictive value to rule out brain injury in mild brain trauma [43]. Both copeptin and PS100 have been shown to correlate positively with the stroke severity [10,44]. The utility of these two markers comes from the idea that copeptin levels rise in the first few hours after endogenous stress and then rapidly fall below normal values [45]. On the other hand, the increase in PS100 is delayed after stroke until tissue necrosis occurs, but the increase is long-lasting [46]. Deboevere et al. (2019) demonstrated that PS100 and copeptin levels were higher in patients who had a stroke compared to those who had other causes of vertigo. Deboevere et al. (2019) [41] concluded that normal levels of copeptin and PS100 exclude the diagnosis of stroke in patients presenting to the emergency room for vertigo. This result is helpful in the diagnosis of stroke, especially in situations of uncertainty or in situations where there is no access to radiology services.

On the other hand, copeptin might be used as a marker in discriminating between stroke and stroke mimics in the pre-hospital setting; this result could be very useful, particularly given that it is a non-invasive test [1,47]. The frequency of stroke mimics can be as high as 30% of stroke code activations at the pre-hospital level [48]. Von Recum et al. (2015) [1] evaluated 36 patients and analyzed the utility of copeptin in the differential diagnosis of ischemic stroke, transient ischemic attack, and stroke mimics (such as complex migraine, delirium, epilepsy, or vestibular neuronitis). Copeptin levels tended to be higher in patients with stroke compared to those with a transient ischemic attack, but, for the patients with stroke mimics, the range of copeptin values was extremely broad [1]. The copeptin level at 4.5 h after the onset of symptoms was two times higher in patients with stroke compared to those with transient ischemic attacks.

Table 1 presents a summary of the findings about copeptin as a marker that helps in the differential diagnosis of stroke, highlighting the number and types of patients included, the assessed markers, the differential diagnoses, and the findings.

## 4. Discussion

One of the first endocrine abnormalities that responds to cerebral ischemia is the activation of the hypothalamic–pituitary–adrenal axis [22].

Our review assessed copeptin as a marker for differential diagnosis in stroke patients. In terms of copeptin levels helping in differentiating between stroke subtypes, the results are inconsistent: some studies did not find any significant differences between cerebral infarction, intracerebral hemorrhage, and subarachnoid hemorrhage [7,29,33]. There was only one study that suggested different correlations between ischemic stroke (positive) and hemorrhagic stroke (negative) [32]. However, all assessed studies explained that copeptin levels can help in differentiating stroke patients from stroke-free patients [7,33].

Other important findings were that the copeptin levels correlate with prognostic scales for several neurological pathologies (NIHSS score, Hunt and Hess score, CGS).

In terms of copeptin levels helping in differentiating between stroke and stroke mimics, the copeptin levels were extremely broad, but the results should be replicated in a greater number of participants. For the diagnosis of vestibular disorders, it was shown that a normal level of copeptin excludes stroke [41].

**Figure 3:**
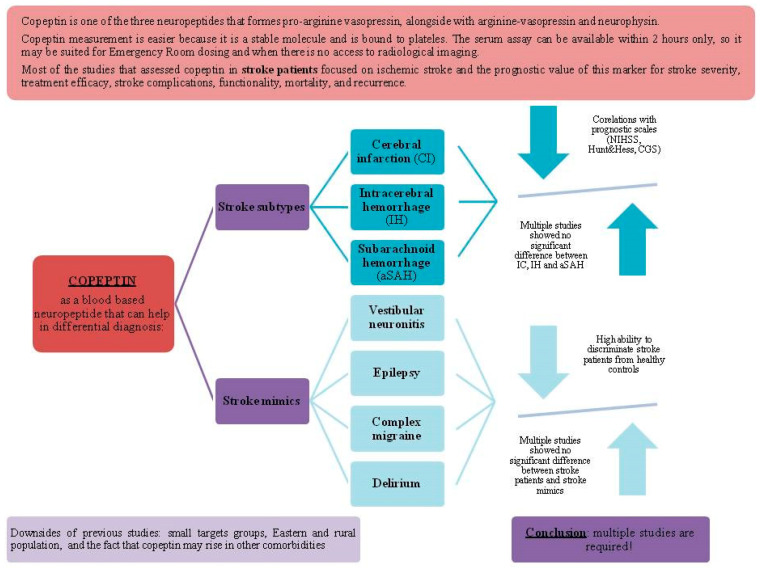
Copeptin use in the differential diagnosis of stroke.

The main findings of the reviewed articles about the differential diagnostic value of copeptin in stroke patients can be seen in Table 2 and Figure 3.

Despite accumulating evidence linking copeptin levels to the outcomes of stroke patients, copeptin measurement is still not routinely performed in the standard management of post-stroke patients [8]. The reliability of these studies is diminished because of three factors: first, the target groups were relatively small; second, most studies were performed in Eastern populations and rural areas; third, copeptin is a marker that can also rise in several other conditions, such as myocardial infarction, polyuria–polydipsia syndrome, hyponatremia, polycystic kidney disease, and metabolic disorders (diabetes mellitus, metabolic syndrome, insulin resistance, infections) [10,12]. However, copeptin levels are elevated in the majority of hyponatremic patients [49]. Copeptin and its relation to hyponatremia are important in stroke patients due to the increased risk of deterioration in the already impaired cerebral circulation [49]. The therapeutic options for hyponatremia include the correction of the underlying cause, fluid restriction, the administration of hypertonic solutions, loop diuretics, and vaptans [49]. For stroke patients, there are no specific guidelines for the treatment of hyponatremia [50,51]. Hyponatremia caused by intracranial diseases should be treated with hypertonic saline [52]. Isotonic saline should be avoided in stroke patients because it can aggravate hyponatremia due to SIADH. Vaptans are approved in the United States because they diminish cerebral edema and the blood–brain barrier through osmotic and anti-inflammatory mechanisms [53,54]. However, studies regarding the role of vaptans in treating stroke patients are still lacking [49]. *Moreover*, the contradictory findings may be explained by the fact that increased AVP levels were found especially in ischemic stroke patients, and they were related to delayed neuronal damage following ischemic and reperfusion, meaning that AVP and copeptin levels may play a more significant role in the longitudinal view of stroke patients, rather than the transverse view, in a single assay, when the patient is at the emergency ward. Other explanations for the contradictory findings may be the heterogeneous and large samples of patients, with wide ranges of NIHSS values, broad age ranges, different stroke etiologies, and different comorbidities among patients.

In line with the limitations of the previous studies, we can formulate some possible future research directions. Copeptin assessment may be useful in acute emergency hospitals with a neurology ward, for patients that present with acute-onset neurological deficits, and may be useful in differentiating between ischemic stroke and hemorrhagic stroke in order to determine the patient’s need for recanalization therapies (the so-called stroke thrombolysis code). Another practical application may be in differentiating between patients who present at the emergency ward for neurological deficits and have no radiological findings on the cerebral CT, in order to triage patients who may suffer from a stroke mimic. The most important consideration in the design of studies on copeptin levels in stroke mimics is to blind the neurologist who interprets the final level of copeptin, as in a previous study that was performed in this field [1]. In addition, future research should focus on the longitudinal follow-up of patients, in order to assess patients who had no imaging findings on cerebral CT but had stroke findings on follow-up MRI or for patient with silent strokes. On the other hand, a copeptin assay may be useful in a multidisciplinary hospital and may be used as an exclusion criterion for stroke in patients who exhibit a sudden altered state of consciousness, thus orienting the clinician toward other metabolic causes of coma. The main future direction is to determine whether copeptin may be used as a marker that directs the diagnosis toward ischemic stroke, similarly to the use of procalcitonine to direct the diagnosis toward sepsis and NT-proBNP to direct the diagnosis toward cardiac insufficiency (although these markers may also be increased in other pathologies, they have high sensitivity and specificity) [55,56]. Another future direction is standardizing the assay of copeptin in terms of when it should be drawn from the patient and how it is stored in order to have the best benefit: future studies should determine the exact time of sampling (for example, for thrombolysis codes, copeptin should be sampled at the same time as the blood samples in line with the inclusion criteria for thrombolysis), as well as precise details of the storage of the blood or plasma sample. Finally, in order to highlight the diagnostic value of copeptin in stroke patients, we suggest that future studies should focus only on this biomarker, as compared to previous studies that analyzed the diagnostic value of multiple biomarkers taken together. In the same context, we suggest that copeptin should be studied in an integration approach with other risk factors or etiological factors related to stroke, as in the CoRisk study [24,57]. Lastly, most of the previous studies focused on determining the plasma level of copeptin, but in the recent years, it was suggested that determining the copeptin level in patient’s cerebrospinal fluid may also help in patients with acute neurological disease, such as subarachnoid hemorrhage [58]. 

## 5. Conclusions

The differential diagnosis of diseases that are presented in the emergency department with acute stroke-like symptoms relies on clinical assessment and brain imaging. However, the limited timeframe in which patients are eligible for recanalization therapies has spurred interest in discovering blood-based markers [48].

The above review suggests that assessing copeptin levels may help in differentiating between stroke subtypes and stroke mimics, but further studies are needed.

Copeptin is a novel and promising marker for the evaluation of cerebrovascular diseases, although multiple diseases can raise copeptin, which may be considered a non-specific marker. Compared to other more specific markers, copeptin is recommended to be used as an additional marker in a multimarker approach [59,60]. Copeptin measurement has the advantage of a rapidly available lab result, in less than 2 h, which makes it useful in emergency situations [10]. It may be used in neurological cases in order to differentiate between stroke subtypes (ischemic versus hemorrhagic). In addition, it may may play an important role in differentiating between stroke and stroke mimics (migraine, vestibular neuronitis, epileptic seizure), but also in differentiating between neurological causes and non-neurological causes of a patient’s symptoms (in comatose patients, for example, who should be admitted to other medical departments). However, because of the small sample sizes of previous studies and the heterogeneous sample populations, further studies are required.

An early diagnosis is critical in stroke patients because of the different and individualized treatments, the usage of acute revascularization therapies, triage decisions, and optimized care [22].

## Figures and Tables

**Table 1:** Studies that evaluated copeptin as a marker for the differential diagnosis of stroke.

Study	Participants	Assessed Markers	Differential Diagnosis	Findings
Aksu et al. (2016) [7]	176 patients before imaging diagnosis	Copeptin	Cerebral infarction, intracerebral hemorrhage, and subarachnoid hemorrhage	- no significant difference between cerebral infarction, intracerebral hemorrhage, and subarachnoid hemorrhage in terms of copeptin level - a difference in copeptin levels between healthy controls and stroke patients
Han et al. (2023) [29]	73 patients (51 with cerebral infarction and 22 with intracerebral hemorrhage)	Copeptin GFAP NT-proBNP	Cerebral infarction and intracerebral hemorrhage	- no significant differences between cerebral infarction and intracerebral hemorrhage in terms of copeptin level
Sun et al. (2018) [32]	119 patients (87 with cerebral infarction and 32 with intracerebral hemorrhage) and 119 healthy controls	Copeptin	Cerebral infarction and intracerebral hemorrhage	- copeptin level was positively associated with cerebral infarction and negatively associated with intracerebral hemorrhage
Sarfo et al. (2018) [33]	156 patients (99 with cerebral infarction and 57 with intracerebral hemorrhage) and 74 healthy controls	Copeptin GFAP MMP-9	Cerebral infarction and intracerebral hemorrhage	- no significant differences between cerebral infarction and intracerebral hemorrhage in any of the markers (for all patients who presented at the emergency room after a mean of 7 days since onset) - when restricting the analysis to the patients who presented to the emergency room in the first 3 days since onset, copeptin was marginally higher among cerebral infarction subjects compared to intracerebral hemorrhage patients - copeptin and GFAP were excellent markers in differentiating between stroke patients and stroke-free patients
Deboevere et al. (2019) [41]	135 patients who presented at the emergency room for vertigo	Copeptin PS100	Central vestibular disorder and peripheral vestibular disorder	- PS100 and copeptin levels were higher in patients who had a stroke compared to those who had other causes of vertigo - normal levels of copeptin and PS100 exclude the diagnosis of stroke in patients presenting to the emergency room for vertigo
Von Recum et al. (2015) [1]	36 patients who presented at the emergency room for stroke-like symptoms	Copeptin	Cerebral infarction, transient ischemic attack, and stroke mimics (such as complex migraine, delirium, epilepsy, or vestibular neuronitis)	- copeptin levels were higher for stroke patients compared to transient ischemic attack patients - patients with stroke mimics had an extremely broad range of copeptin values

**Table 2:** Main findings of studies that evaluated the differential diagnostic value of copeptin in stroke patients.

Study	Main Findings
Aksu et al. (2016) [7]	No significant correlation between copeptin level and ICH score for hemorrhagic stroke. Significant correlation between copeptin level and NIHSS score for ischemic stroke. Significant correlation between copeptin level and Hunt and Hess score for subarachnoid hemorrhage. Significant correlation between copeptin level and CGS. Copeptin levels oriented toward the patient who died and those who survived. Copeptin levels oriented toward healthy volunteers and those with neurological disease. The authors did not conclude that copeptin can differentiate between ischemic stroke, hemorrhagic stroke, and subarachnoid hemorrhage.
Han et al. (2023) [29]	The authors did not conclude that copeptin can differentiate between ischemic stroke and hemorrhagic stroke.
Sun et al. (2018) [32]	Copeptin levels oriented toward healthy volunteers and those with neurological disease. Copeptin levels can differentiate between ischemic stroke and hemorrhagic stroke.
Sarfo et al. (2018) [33]	Copeptin levels oriented toward healthy volunteers and those with neurological disease. The authors did not conclude that copeptin can differentiate between ischemic stroke and hemorrhagic stroke (for patients with late onset). The authors concluded that copeptin may differentiate between ischemic stroke and hemorrhagic stroke (for patients with early onset). Copeptin levels did not help in differentiating between stroke causes (atherothrombotic, cardioembolic, and lacunar strokes).

## Data Availability

No new data were created or analyzed in this study. Data sharing is not applicable to this article.

## References

[1] Von Recum J., Searle J., Slagman A., Vollert J.O., Endres M., Möckel M., Ebinger M. (2015). Copeptin: Limited Usefulness in Early Stroke Differentiation?. Stroke Res. Treat..

[2] Chai S.B., Hui Y.M., Li X.M., Xiao Y., Tang C.S. (2009). Plasma Levels of Copeptin in Patients with Coronary Heart Disease. Heart Vessel..

[3] Khan S.Q., Dhillon O.S., O’Brien R.J., Struck J., Quinn P.A., Morgenthaler N.G., Squire I.B., Davies J.E., Bergmann A., Ng L.L. (2007). C-Terminal Provasopressin (Copeptin) as a Novel and Prognostic Marker in Acute Myocardial Infarction: Leicester Acute Myocardial Infarction Peptide (LAMP) Study. Circulation.

[4] Voors A.A., von Haehling S., Anker S.D., Hillege H.L., Struck J., Hartmann O., Bergmann A., Squire I., van Veldhuisen D.J., Dickstein K. (2009). C-Terminal Provasopressin (Copeptin) Is a Strong Prognostic Marker in Patients with Heart Failure after an Acute Myocardial Infarction: Results from the OPTIMAAL Study. Eur. Heart J..

[5] Westermann I., Dünser M.W., Haas T., Jochberger S., Luckner G., Mayr V.D., Wenzel V., Stadlbauer K.-H., Innerhofer P., Morgenthaler N. (2007). Endogenous Vasopressin and Copeptin Response in Multiple Trauma Patients. Shock.

[6] Yu G.-F., Huang Q., Dai W.-M., Jie Y.-Q., Fan X.-F., Wu A., Lv Y., Li Y.-P., Yan X.-J. (2012). Prognostic Value of Copeptin: One-Year Outcome in Patients with Traumatic Brain Injury. Peptides.

[7] Aksu F., Gurger M., Yilmaz M., Atescelik M., Yildiz M., Ilhan N., Ilhan S., Goktekin M.C. (2016). Copeptin Levels in Cerebral Infarction, Intracranial Hemorrhage and Subarachnoid Hemorrhage. Clin. Lab..

[8] Blek N., Szwed P., Putowska P., Nowicka A., Drela W.L., Gasecka A., Ladny J.R., Merza Y., Jaguszewski M.J., Szarpak L. (2022). The Diagnostic and Prognostic Value of Copeptin in Patients with Acute Ischemic Stroke and Transient Ischemic Attack: A Systematic Review and Meta-Analysis. Cardiol. J..

[9] Choi K.-S., Kim H.J., Chun H.-J., Kim J.M., Yi H.-J., Cheong J.-H., Kim C.-H., Oh S.-J., Ko Y., Kim Y.-S. (2015). Prognostic Role of Copeptin after Stroke: A Systematic Review and Meta-Analysis of Observational Studies. Sci. Rep..

[10] Christ-Crain M. (2019). Vasopressin and Copeptin in Health and Disease. Rev. Endocr. Metab. Disord..

[11] Montellano F.A., Ungethüm K., Ramiro L., Nacu A., Hellwig S., Fluri F., Whiteley W.N., Bustamante A., Montaner J., Heuschmann P.U. (2021). Role of Blood-Based Biomarkers in Ischemic Stroke Prognosis. Stroke.

[12] Baranowska B., Kochanowski J. (2019). Copeptin-a New Diagnostic and Prognostic Biomarker in Neurological and Cardiovascular Diseases. Neuroendocrinol. Lett..

[13] Morgenthaler N.G. (2010). Copeptin: A Biomarker of Cardiovascular and Renal Function. Congest. Heart Fail..

[14] Enhörning S., Hedblad B., Nilsson P.M., Engström G., Melander O. (2015). Copeptin Is an Independent Predictor of Diabetic Heart Disease and Death. Am. Heart J..

[15] Barreca T., Gandolfo C., Corsini G., Del Sette M., Cataldi A., Rolandi E., Franceschini R. (2001). Evaluation of the Secretory Pattern of Plasma Arginine Vasopressin in Stroke Patients. Cerebrovasc. Dis..

[16] Greisenegger S., Segal H.C., Burgess A.I., Poole D.L., Mehta Z., Rothwell P.M. (2015). Copeptin and Long-Term Risk of Recurrent Vascular Events after Transient Ischemic Attack and Ischemic Stroke: Population-Based Study. Stroke.

[17] Morgenthaler N.G., Struck J., Alonso C., Bergmann A. (2006). Assay for the Measurement of Copeptin, a Stable Peptide Derived from the Precursor of Vasopressin. Clin. Chem..

[18] Nigro N., Müller B., Morgenthaler N.G., Fluri F., Schuetz P., Neiderta S., Stolz D., Bingisser R., Tamm M., Christ-Crain M. (2011). The Use of Copeptin, the Stable Peptide of the Vasopressin Precursor, in the Differential Diagnosis of Sodium Imbalance in Patients with Acute Diseases. Swiss Med. Wkly..

[19] Bhandari S.S., Loke I., Davies J.E., Squire I.B., Struck J., Ng L.L. (2009). Gender and Renal Function Influence Plasma Levels of Copeptin in Healthy Individuals. Clin. Sci..

[20] Dobsa L., Cullen Edozien K. (2013). Copeptin and Its Potential Role in Diagnosis and Prognosis of Various Diseases. Biochem. Med..

[21] Potocki M., Breidthardt T., Mueller A., Reichlin T., Socrates T., Arenja N., Reiter M., Morgenthaler N.G., Bergmann A., Noveanu M. (2010). Copeptin and Risk Stratification in Patients with Acute Dyspnea. Crit. Care.

[22] Zhang J.-L., Yin C.-H., Zhang Y., Zhao L.-B., Fu H.-J., Feng J.-C. (2013). Plasma Copeptin and Long-Term Outcomes in Acute Ischemic Stroke. Acta Neurol. Scand..

[23] Săcărescu A., Turliuc M.-D., Brănișteanu D.D. (2021). Role of Copeptin in the Diagnosis of Traumatic Neuroendocrine Dysfunction. Neuropeptides.

[24] De Marchis G.M., Katan M., Weck A., Fluri F., Foerch C., Findling O., Schuetz P., Buhl D., El-Koussy M., Gensicke H. (2013). Copeptin Adds Prognostic Information after Ischemic Stroke: Results from the CoRisk Study. Neurology.

[25] Vasile A.I., Tiu C., Badiu C. (2024). Copeptin as Biomarker for Acute Ischemic Stroke Prognosis and Revascularization Treatment Efficacy. Front. Neurol..

[26] Azhari H.F. (2024). Advancing Stroke Diagnosis and Management through Nuclear Medicine: A Systematic Review of Clinical Trials. Front. Med..

[27] Katan M., Fluri F., Morgenthaler N.G., Schuetz P., Zweifel C., Bingisser R., Müller K., Meckel S., Gass A., Kappos L. (2009). Copeptin: A Novel, Independent Prognostic Marker in Patients with Ischemic Stroke. Ann. Neurol..

[28] Moher D., Liberati A., Tetzlaff J., Altman D.G., The PRISMA Group (2009). Preferred Reporting Items for Systematic Reviews and Meta-Analyses: The PRISMA Statement. PLoS Med..

[29] Han E., Kim H., Cho B., Lee J.-J., Shin S., Oh E.-J., Chae H. (2023). Plasma Glial Fibrillary Acidic Protein and N-Terminal Pro B-Type Natriuretic Peptide: Potential Biomarkers to Differentiate Ischemic and Hemorrhagic Stroke. Diagnostics.

[30] Eng L.F., Ghirnikar R.S., Lee Y.L. (2000). Glial Fibrillary Acidic Protein: GFAP-Thirty-One Years (1969–2000). Neurochem. Res..

[31] Brunkhorst R., Pfeilschifter W., Foerch C. (2010). Astroglial Proteins as Diagnostic Markers of Acute Intracerebral Hemorrhage—Pathophysiological Background and Clinical Findings. Transl. Stroke Res..

[32] Sun H., Huang D., Wang H., Zhou B., Wu X., Ma B., Shi J. (2018). Association between Serum Copeptin and Stroke in Rural Areas of Northern China: A Matched Case-Control Study. Dis. Markers.

[33] Sarfo F.S., Owusu D., Adamu S., Awuah D., Appiah L., Amamoo M., Loglo A., Owolabi M., Ovbiagele B. (2018). Plasma Glial Fibrillary Acidic Protein, Copeptin, and Matrix Metalloproteinase-9 Concentrations among West African Stroke Subjects Compared with Stroke-Free Controls. J. Stroke Cerebrovasc. Dis..

[34] Zhang X., Lu X.-M., Huang L.-F., Ye H. (2012). Copeptin Is Associated with One-Year Mortality and Functional Outcome in Patients with Acute Spontaneous Basal Ganglia Hemorrhage. Peptides.

[35] Zweifel C., Katan M., Schuetz P., Siegemund M., Morgenthaler N.G., Merlo A., Mueller B., Christ-Crain M. (2010). Copeptin Is Associated with Mortality and Outcome in Patients with Acute Intracerebral Hemorrhage. BMC Neurol..

[36] Galea J., Ogungbenro K., Hulme S., Patel H., Scarth S., Hoadley M., Illingworth K., McMahon C.J., Tzerakis N., King A.T. (2017). Reduction of Inflammation after Administration of Interleukin-1 Receptor Antagonist Following Aneurysmal Subarachnoid Hemorrhage: Results of the Subcutaneous Interleukin-1Ra in SAH (SCIL-SAH) Study. J. Neurosurg..

[37] Vergouwen M.D., Vermeulen M., van Gijn J., Rinkel G.J., Wijdicks E.F., Muizelaar J.P., Mendelow A.D., Juvela S., Yonas H., Terbrugge K.G. (2010). Definition of Delayed Cerebral Ischemia after Aneurysmal Subarachnoid Hemorrhage as an Outcome Event in Clinical Trials and Observational Studies: Proposal of a Multidisciplinary Research Group. Stroke.

[38] Zuo Z., Ji X. (2019). Prognostic Value of Copeptin in Patients with Aneurysmal Subarachnoid Hemorrhage. J. Neuroimmunol..

[39] Fung C., de Marchis G.M., Katan M., Seiler M., Arnold M., Gralla J., Raabe A., Beck J. (2013). Copeptin as a Marker for Severity and Prognosis of Aneurysmal Subarachnoid Hemorrhage. PLoS ONE.

[40] Zhu X.-D., Chen J.-S., Zhou F., Liu Q.-C., Chen G., Zhang J.-M. (2011). Detection of Copeptin in Peripheral Blood of Patients with Aneurysmal Subarachnoid Hemorrhage. Crit. Care.

[41] Deboevere N., Marjanovic N., Sierecki M., Marchetti M., Dubocage M., Magimel E., Mimoz O., Guenezan J. (2019). Value of Copeptin and the S-100b Protein Assay in Ruling out the Diagnosis of Stroke-Induced Dizziness Pattern in Emergency Departments. Scand. J. Trauma Resusc. Emerg. Med..

[42] Beaudeux J.L., Soler C., Foglietti M.J. (2002). Physiopathologie de La Protéine S-100 β: Intérêt de Son Dosage En Biologie Clinique. Immuno-Anal. Biol. Spéc..

[43] Undén L., Calcagnile O., Undén J., Reinstrup P., Bazarian J. (2015). Validation of the Scandinavian Guidelines for Initial Management of Minimal, Mild and Moderate Traumatic Brain Injury in Adults. BMC Med..

[44] Nash D.L., Bellolio M.F., Stead L.G. (2008). S100 as a Marker of Acute Brain Ischemia: A Systematic Review. Neurocrit. Care.

[45] Szmydynger-Chodobska J., Fox L.M., Lynch K.M., Zink B.J., Chodobski A. (2010). Vasopressin Amplifies the Production of Proinflammatory Mediators in Traumatic Brain Injury. J. Neurotrauma.

[46] Purrucker J.C., Herrmann O., Lutsch J.K., Zorn M., Schwaninger M., Bruckner T., Auffarth G.U., Veltkamp R. (2014). Serum Protein S100β Is a Diagnostic Biomarker for Distinguishing Posterior Circulation Stroke from Vertigo of Nonvascular Causes. Eur. Neurol..

[47] Wendt M., Ebinger M., Kunz A., Rozanski M., Waldschmidt C., Weber J.E., Winter B., Koch P.M., Nolte C.H., Hertel S. (2015). Copeptin Levels in Patients with Acute Ischemic Stroke and Stroke Mimics. Stroke.

[48] Hand P.J., Kwan J., Lindley R.I., Dennis M.S., Wardlaw J.M. (2006). Distinguishing between Stroke and Mimic at the Bedside: The Brain Attack Study. Stroke.

[49] Liamis G., Barkas F., Megapanou E., Christopoulou E., Makri A., Makaritsis K., Ntaios G., Elisaf M., Milionis H. (2019). Hyponatremia in Acute Stroke Patients: Pathophysiology, Clinical Significance, and Management Options. Eur. Neurol..

[50] Verbalis J.G., Goldsmith S.R., Greenberg A., Korzelius C., Schrier R.W., Sterns R.H., Thompson C.J. (2013). Diagnosis, Evaluation, and Treatment of Hyponatremia: Expert Panel Recommendations. Am. J. Med..

[51] Spasovski G., Vanholder R., Allolio B., Annane D., Ball S., Bichet D., Decaux G., Fenske W., Hoorn E.J., Ichai C. (2014). Clinical Practice Guideline on Diagnosis and Treatment of Hyponatraemia. Eur. J. Endocrinol..

[52] Sterns R.H., Silver S.M. (2008). Cerebral Salt Wasting Versus SIADH: What Difference?. J. Am. Soc. Nephrol..

[53] Schrier R.W., Gross P., Gheorghiade M., Berl T., Verbalis J.G., Czerwiec F.S., Orlandi C. (2006). Tolvaptan, a Selective Oral Vasopressin V_2_-Receptor Antagonist, for Hyponatremia. N. Engl. J. Med..

[54] Zeynalov E., Jones S. (2016). Recent Advances and Future Directions in Preclinical Research of Arginine-Vasopressin (AVP) Receptor Blocker Conivaptan in the Context of Stroke. Neural Regen. Res..

[55] Cao Z., Jia Y., Zhu B. (2019). BNP and NT-proBNP as Diagnostic Biomarkers for Cardiac Dysfunction in Both Clinical and Forensic Medicine. Int. J. Mol. Sci..

[56] Mućka S., Jakubiak G.K., Pawlas N. (2025). Procalcitonin: Infection or Maybe Something More? Noninfectious Causes of Increased Serum Procalcitonin Concentration: Updated Knowledge. Life.

[57] De Marchis G.M., Dankowski T., König I.R., Fladt J., Fluri F., Gensicke H., Foerch C., Findling O., Kurmann R., Fischer U. (2019). A Novel Biomarker-Based Prognostic Score in Acute Ischemic Stroke: The CoRisk Score. Neurology.

[58] Mindt S., Andrade-Barazarte H., Tokhi U., Ludtka C., Neumaier M., Hänggi D. (2019). Immunoluminometric assay for copeptin measurement in cerebrospinal fluid: Technical aspects and pilot study. Clin. Chim. Acta.

[59] Ion A., Stafie C., Mitu O., Ciobanu C.E., Halitchi D.I., Costache A.D., Bobric C., Troase R., Mitu I., Huzum B. (2021). Biomarkers Utility: At the Borderline between Cardiology and Neurology. J. Cardiovasc. Dev. Dis..

[60] Möckel M., Searle J. (2014). Copeptin—Marker of Acute Myocardial Infarction. Curr. Atheroscler. Rep..

